# Supercurrent and Superconducting Diode Effect in Parallel Double Quantum Dots with Rashba Spin–Orbit Interaction

**DOI:** 10.3390/ma17184497

**Published:** 2024-09-13

**Authors:** Feng Chi, Yaohong Shen, Yumei Gao, Jia Liu, Zhenguo Fu, Zichuan Yi, Liming Liu

**Affiliations:** 1 School of Electronic and Information Engineering, UEST of China, Zhongshan Institute, Zhongshan 528400, China; chifeng@semi.ac.cn (F.C.); yumeigao@zsc.edu.cn (Y.G.); yizichuan@zsc.edu.cn (Z.Y.); liulmxps@zsc.edu.cn (L.L.); 2 South China Academy of Advanced Optoelectronics, South China Normal University, Guangzhou 510006, China; 2023024194@m.scnu.edu.cn; 3 School of Science, Inner Mongolia University of Science and Technology, Baotou 014010, China; jialiu@imust.edu.cn; 4 Institute of Applied Physics and Computational Mathematics, Beijing 100088, China

**Keywords:** supercurrent, superconducting diode effect, parallel double quantum dots, spin–orbit interaction, magnetic flux

## Abstract

We study theoretically the supercurrent and the superconducting diode effect (SDE) in a structure comprising parallel-coupled double quantum dots (DQDs) sandwiched between two superconductor leads in the presence of a magnetic flux. The influence of the Rashba spin–orbit interaction (RSOI), which induces a spin-dependent phase factor in the dot–superconductor coupling strength, is taken into account by adopting the nonequilibrium Green’s function technique. This RSOI-induced phase factor serves as a driving force for the supercurrent in addition to the usual superconducting phase difference, and it leads to the system’s left/right asymmetry. Correspondingly, the magnitude of the positive and negative critical currents become different from each other: the so-called SDE. Our results show that the period, magnitude, and direction of the supercurrents depend strongly on the RSOI-induced phase factor, dots’ energy levels, interdot coupling strengths, and the magnetic flux. In the absence of magnetic flux, the diode efficiency is negative and may approach −2, which indicates the perfect diode effect with only negative flowing supercurrent in the absence of a positive one. Interestingly enough, both the sign and magnitude of the diode efficiency can be efficiently adjusted with the help of magnetic flux, the dots’ energy levels and the interdot coupling strength and thus provide a controllable SDE by rich means, such as gate voltage or host materials of the system.

## 1. Introduction

The supercurrent refers to the equilibrium current that occurs when the phases of two superconductors, which are separated by a metallic [1] or a thin insulating material [2], are different from each other in the absence of bias voltage. This phenomenon is known as the DC Josephson effect that originated from the superconducting phase difference or phase bias [2], and the relevant superconducting electronics have gained growing renewed interest in recent years due to their fundamental research values and wide applications [3,4]. The supercurrent is carried by the Andreev bound states formed within the superconducting gap, and it is usually an odd sine-like function with respect to the phase bias $\varphi =\varphi_{L}-\varphi_{R}$, where $\varphi_{L/R}$ is the superconducting phase in the left/right superconductor. The superconducting quantum interference devices (SQUIDs) can be used to detect tiny variations in magnetic fields, and they have been widely used in various fields, including medical and industrial instruments [2]. In recent years, superconducting electronics consisting of Josephson junctions were proposed to construct quantum computers [3], medical diagnostic devices [4,5], extremely sensitive magnetometers [6] and voltmeters [7] as well as spintronic devices [8].

If the absolute values of positive and negative critical currents, which are individually the maximum and minimum supercurrents flowing in opposite directions within one period of the phase bias φ, are not equal to each other, the supercurrent becomes orientation-dependent, which is a phenomenon called the superconducting diode effect (SDE) [4,9,10,11,12,13,14]. It plays a key role in superconducting electronics and is in analogy to the semiconductor diode with direction-dependent electrical resistance. The SDE characterized by nonreciprocal supercurrents was first realized in SQUIDs based on superconducting bridges [15] and tunnel Josephson junctions [16]. It was then observed in superconducting thin films’ lack of geometric symmetries [17]. In recent years, the SDE was theoretically proposed and experimentally realized in diverse platforms, including superconducting thin films without junctions [18,19], Josephson junctions based on semiconductors with spin–orbit coupling [20,21], or materials with intrinsic asymmetries in the absence of magnetic fields [22]. Another kind of platform focuses on achieving the SDE with the help of a quantum interference effect, where nonsinusoidal current-phase relations with a period larger than the conventional 2π are generated in devices with multiple transport paths [23]. The supercurrent in such an SDE interferometer is partially due to the different harmonic content between different paths and a magnetic flux penetrating through the loop [24], as was recently demonstrated in experiments [25], in which large diode efficiencies up to approximately 30% were reported.

To explore more novel supercurrents and the SDE, researchers have tried to insert various materials between the superconductors, of which one is the semiconductor quantum dots (QDs) with fully controllable quantized energy levels, interactions to environments, confined carriers in them, etc. [26]. Hybridized superconductor/QDs structures have been realized, and the supercurrent carried by Andreev bound states was studied [27,28,29,30,31,32,33,34]. It was shown that the QDs act as controllable magnetic impurities, and the confined electron in them can couple to quasiparticles in the superconductors, inducing various states within the superconducting gap. The magnitude, flowing directions, and period of the supercurrent tunneling through such systems are then fully manipulated by adjusting parameters related to the QDs. Recently, SDE [35,36] and spin SDE [37] with high diode efficiencies were proposed in QDs connected to conventional superconductors in the presence of magnetic impurity or spin–orbit interaction. If the superconductor leads are driven by a topological state, the exotic Majorana bound states may emerge in them, which changes the supercurrent drastically [38,39,40,41,42]. In particular, double QDs (DQDs) arranged in a parallel configuration were proposed theoretically to be embedded between two superconductor leads [32,43,44]. It was found that the supercurrent depends strongly on the quantum interference effects originated from the two transport paths through the QDs and the applied magnetic flux. Very recently, such structures with strong Rashba spin–orbit interaction (RSOI) in the QDs were successfully realized in experiments [45,46,47]. Interesting phenomena including the π−0 transition, dependence on the spin state, and orbital hybridization, as well as interdot couplings of the supercurrent, were found.

**Figure 1:**
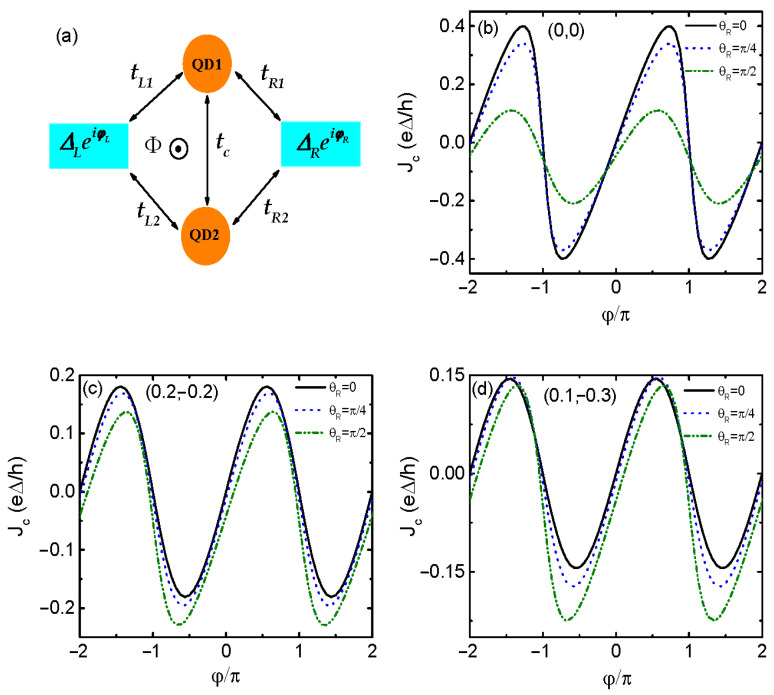
(**a**) Schematic plot of the parallel DQDs coupled to the left and right superconductor leads having energy gap $\Delta_{L/R}$ and phase factor $\varphi_{L/R}$. The dot-lead and interdot couplings indicated by the black lines with double sided arrows are denoted by $t_{\alpha i}$ and $t_{c}$, respectively. The RSOI in the QDs induces a spin-dependent phase factor in $t_{\alpha i}$ and is responsible for the SDE in the present device. We also consider that a magnetic flux Φ is applied on the system and changes both the amplitude and sign of the diode efficiency. (**b**–**d**) are the supercurrent varying as a function of the superconducting phase difference $\varphi =\varphi_{L}-\varphi_{R}$ and different values of RSOI-induced phase factor $\theta_{R}$ for dots’ energy levels configurations of (0,0), (0.2,−0.2) and (0.1,−0.3), respectively. The interdot coupling strength is fixed at $t_{c}=0.1$ and magnetic flux ϕ=0.

In the experiment work of Refs. [46,47], the authors mainly discussed the influences of spin states and orbital hybridization related to the RSOI on the supercurrents. In fact, the RSOI in such systems will induce a spin-dependent phase factor in the hybridization between the QDs and the leads [48,49,50,51], which will change the supercurrent drastically. Moreover, although the authors have considered the impacts of magnetic fields on the supercurrent, possible functions of the magnetic flux, which induces an interesting quantum interference effect, were omitted. In view of those previous theoretical and experimental work, here, we study properties of supercurrents in parallel DQDs inserted between two superconductor leads, as shown in Figure 1a, taking both the phase factors induced by RSOI and magnetic flux penetrating through the system into consideration. Furthermore, we pay special attention to the RSOI-induced SDE, which has not been touched in such a system yet. Our results show that the RSIO-induced phase factors may act as a driving force for the supercurrent in addition to the phase bias from the superconductors, and it also induces the structure’s left/right asymmetry, which is responsible for the emergence of the SDE. We find that the diode efficiency depends on the dots’ energy levels, interdot coupling and the magnetic flux. By the joint action of the RSOI-induced phase factor and the interdot coupling strength, the critical current can flow only in one direction, and the current of the opposite direction is suppressed to zero, showing a perfect diode effect. More interestingly, the direction of the critical current can be reversed with the help of magnetic flux. It is well known that the magnetic field will bring about various interesting phenomena, including the magnetic flux, Zeeman splitting of the energy levels, and optical absorbance under time-dependent magnetic fields. In the present paper, we focus on the impacts of the magnetic flux because it is crucial for the interesting diode effect. If the magnetic field is applied in the *x* or *y* direction, other interesting phenomena may be generated by the inter-level spin-flip effects, as was demonstrated in Refs. [36,44,45,46,47,52]. The advantages of the present parallel DQDs include the tunable discrete energy levels of the QDs, interdot couplings and the two electron transport paths for the quantum interference effect. As is compared to the multiple-path system, ref. [53] disadvantages of the present device include the optical absorbance and sensitive magnetoplasmonic interactions via the magnetic field as were demonstrated Ref. [53].

## 2. Model and Method

The present Josephson junction consists of two QDs arranged in a parallel configuration that are coupled to the left and right superconductor leads, as shown in Figure 1a. For simplicity, we consider that there is only one energy level in each QD, and the general Hamiltonian can be written as $H=H_{DQDs}+H_{leads}+H_{T}$ [29,30,31,43,44,48,49], in which the Hamiltonian of the DQDs and interaction between them is

(1) $$\begin{array}{c} H_{DQDs}=\sum_{i,\sigma }\varepsilon_{i}d_{i\sigma }^{†}d_{i\sigma }+t_{c}\sum_{\sigma }\left(d_{1\sigma }^{†}d_{2\sigma }+d_{2\sigma }^{†}d_{1\sigma }\right), \end{array}$$

where the creation (annihilation) operator $d_{i\sigma }^{†}$$\left(d_{i\sigma }\right)$ is for electrons in dot-*i* with energy level $\varepsilon_{i}$ and spin state σ= ↑,↓. $\varepsilon_{i}$ is tunable in experiments by gate voltages $V_{g}$ as $\varepsilon_{i}=\varepsilon_{i,0}-eV_{g}$ with $\varepsilon_{i,0}$ representing the bare energy level in dot-*i*. The direct tunnel coupling strength between the DQDs is $t_{c}$. The parallel DQDs are usually coupled by a tunnel junction, as demonstrated by the experiments of Refs. [45,46,47]. The coupling strength between the quantum dots depends on the distance between the quantum dots, the material of the junction, and the shape and size of the junction that can be changed by the gate voltage. The Hamiltonian $H_{leads}$ stands for the left and right superconductor leads coupled to the DQDs, which is given by [29,30]

(2) $$\begin{array}{c} H_{leads}=\sum_{\alpha ,k,\sigma }\varepsilon_{\alpha ,k\sigma }C_{\alpha ,k\sigma }^{†}C_{\alpha ,k\sigma }+\sum_{\alpha ,k}\left(\Delta_{\alpha }e^{i\varphi_{\alpha }}C_{\alpha ,k↑}^{†}C_{\alpha ,-k↓}+H.c.\right) \end{array}$$

where $C_{\alpha ,k\sigma }^{†}\left(C_{\alpha ,k\sigma }\right)$ is the creation (annihilation) operator of the electron in lead α(α=L,R) with energy $\varepsilon_{\alpha ,k\sigma }$, superconducting energy gap $\Delta_{\alpha }$ and phase $\varphi_{\alpha }$. In the present manuscript, we study the supercurrent arisen from the phase difference $\varphi_{L}-\varphi_{R}$ in the absence of external bias voltage, and we set the chemical potentials of the left and right leads as $\mu_{L}=\mu_{R}=0$. The Hamiltonian $H_{T}$ is for the tunneling between the DQDs and the leads, whose explicit expression is [29,30]

(3) $$\begin{array}{c} H_{T}=\sum_{\alpha ,k,i,\sigma }\left(t_{\alpha i\sigma }C_{\alpha ,k\sigma }^{†}d_{i\sigma }+H.c.\right) \end{array}$$

where $t_{\alpha i\sigma }$ is the coupling strength between dot-*i* and lead-α. Due to the existence of the perpendicular magnetic field $B_{z}$, a phase ϕ is added in the dot-lead hopping elements, and $\phi =\int \vec{A}\cdot d\vec{r}/\phi_{0}$ with the vector potential $\vec{A}=\left(-By,0,0\right)$ and $\phi_{0}=\hbar /e$. The Zeeman splitting of the QDs’ energy levels is neglected in the present paper, as it essentially does not change the obtained results. Taking the phase factors ϕ and $\theta_{Ri}$ individually arisen from the magnetic flux Φ and RSOI in QD-*i* into consideration, the explicit expressions of $t_{\alpha i\sigma }$ are written as $t_{L1\sigma }=\left|t_{L1}\right|e^{i\phi /4}e^{i\sigma \theta_{R1}}$, $t_{L2\sigma }=\left|t_{L2}\right|e^{-i\phi /4}e^{i\sigma \theta_{R2}}$, $t_{R1\sigma }=\left|t_{R1}\right|e^{-i\phi /4}$, and $t_{R2\sigma }=\left|t_{R2}\right|e^{i\phi /4}$ [43,44,48,49].

The supercurrent $J_{c}$ can be calculated from the evolution of the particle number operator of the electrons in the superconductor leads, and it is given in terms of the Green’s functions of the DQDs as [29,30,38,43,44].

(4) Jc=Jc↑+Jc↓=2eħ∫dεReTr[Gda(ΣLa−ΣRa)−Gdr(ΣLr−ΣRr)]f(ε),

where ${\tilde{\sigma }}_{z}=\mathrm{diag}\left(1,-1,1,-1,1,-1,1,-1\right)$ is a 8×8 diagonal matrix, $G_{d}^{r/a}$ is the retarded/advanced Green’s function of the DQDs, and $\Sigma_{L/R}^{r/a}$ is the retarded/advanced self-energy contributed from the left/right superconductor lead. The quantity $f\left(\varepsilon \right)=1/[1+\exp\left(\varepsilon /k_{B}T\right)]$ is the equilibrium Dirac–Fermi function with *T* and $k_{B}$ representing the temperature and Boltzmann constant, respectively. In the following, we adopt the Dyson equation method to calculate the dots’ Green’s functions. To proceed, we first divide the system into four parts, which are dot-1, dot-2, the left lead, and the right lead. Then, we rewrite the system Hamiltonian in a 16×16 matrix form as $H=\frac{1}{2}\Psi^{†}H\Psi$ in the basis of $\Psi =(\Psi_{d1},\Psi_{d2},\Psi_{L},\Psi_{R})$ with the four sub-basis of $\Psi_{\beta }={\left(\psi_{\beta ↑}^{†},\psi_{\beta ↓},\psi_{\beta ↓}^{†},\psi_{\beta ↓}\right)}^{†}$, in which β=d1,d2,L,R, respectively. The transformed Hamiltonian H is given by

(5) $$\begin{array}{c} H=\left[\begin{array}{cccc} \;\;H_{d1} & H_{d1,d2} & H_{d1,L} & H_{d1,R}\\ \;\;H_{d2,d1} & H_{d2} & H_{d2,L} & H_{d2,R}\\ \;\;H_{L,d1} & H_{L,d2} & H_{L} & 0\\ \;\;H_{R,d1} & H_{R,d2} & 0 & H_{R} \end{array}\right], \end{array}$$

in which the 4×4 sub-matrix $H_{di}=$ diag$\left(\varepsilon_{i},-\varepsilon_{i},\varepsilon_{i},-\varepsilon_{i}\right)$, $H_{d1,d2}=H_{d2,d1}^{†}=$ diag$\left(t_{c},-t_{c},t_{c},-t_{c}\right)$, $H_{di,\alpha }=H_{\alpha ,di}^{†}=$ diag$\left(t_{\alpha i↑}^{*},-t_{\alpha i↓},t_{\alpha i↓}^{*},-t_{\alpha i↑}\right)$, and [35,43,44]

(6) $$\begin{array}{c} H_{\alpha =L,R}=-i\pi \rho_{\alpha }\left(\varepsilon \right)\gamma_{\alpha }\left(\varepsilon \right)\left[\begin{array}{cccc} \;\;1 & -\frac{\Delta_{\alpha }}{\varepsilon }e^{-i\varphi } & 0 & 0\\ \;\;-\frac{\Delta_{\alpha }}{\varepsilon }e^{i\varphi } & 1 & 0 & 0\\ \;\;0 & 0 & 1 & \frac{\Delta_{\alpha }}{\varepsilon }e^{-i\varphi }\\ \;\;0 & 0 & \frac{\Delta_{\alpha }}{\varepsilon }e^{i\varphi } & 1 \end{array}\right], \end{array}$$

where $\rho_{\alpha }\left(\varepsilon \right)$ is the normal density of states of lead-α and is independent of the energy variable under wide-band approximation. The factor $\gamma_{\alpha }\left(\varepsilon \right)$ is defined as

(7) $$\begin{array}{c} \gamma_{\alpha }\left(\varepsilon \right)=\frac{\left|\varepsilon |\vartheta (|\varepsilon |-\Delta_{\alpha }\right)}{\sqrt{\varepsilon^{2}-\Delta_{\alpha }^{2}}}+\frac{\varepsilon \vartheta (\Delta_{\alpha }-|\varepsilon |)}{i\sqrt{\Delta_{\alpha }^{2}-\varepsilon^{2}}}, \end{array}$$

where ϑ(x)=1 for x>0 and ϑ(x)=0 otherwise. The retarded/advanced Green’s function in Equation (4) then is obtained with the help of the Dyson equation,

(8) $$\begin{array}{c} G^{r}=g^{r}+g^{r}\Sigma^{r}G^{r}, \end{array}$$

in which $g^{r}$ is the retarded Green’s function of the DQDs and the interaction between them, and $\Sigma^{r}$ represents the self-energy due to couplings between the DQDs and the leads. The 8×8 matrix $g^{r}$ is given by [35,38]

(9) $$\begin{array}{c} g^{r}={\left[\begin{array}{cc} \;\;\varepsilon -H_{d1}+i0^{+} & -H_{d1,d2}\\ \;\;\;\;-H_{d2,d1} & \varepsilon -H_{d2}+i0^{+} \end{array}\right]}^{-1}. \end{array}$$

The self-energy in Equation (8) is $\Sigma^{r}=\Sigma_{L}^{r}+\Sigma_{R}^{r}$, in which

(10) $$\begin{array}{c} \Sigma_{\alpha }^{r}={\left[\begin{array}{cc} \;\;\Sigma_{\alpha ;11}^{r} & \Sigma_{\alpha ;12}^{r}\\ \;\;\Sigma_{\alpha ;21}^{r} & \Sigma_{\alpha ;22}^{r} \end{array}\right]}^{-1}, \end{array}$$

where the 4×4 sub-matrix is calculated by $\Sigma_{\alpha ;ij}^{r}=H_{di,\alpha }H_{\alpha }H_{\alpha ,dj}$ with α=L,R; i,j=1,2 and $\Sigma_{\alpha ;ij}^{a}={\left(\Sigma_{\alpha ;ij}^{r}\right)}^{†}$ [43,44]. In the self-energy, we need to define the linewidth function $\Gamma_{\alpha i}=-2i\pi \rho \left(\varepsilon \right){\left|t_{\alpha i}\right|}^{2}$ describing coupling between dot-*i* and lead-α. The diode efficiency of the SDE is given by $\eta =(J_{c+}-|J_{c-}\left|\right)/J_{cm}$ [35,36,37], where $J_{cm}=(J_{c+}-|J_{c-}\left|\right)/2$ is the mean critical supercurrent with $J_{c+}$ ($J_{c-}$) flowing in the positive (negative) direction.

## 3. Numerical Results

In this section, we present the supercurrent and its diode effect varying with respect to superconducting phase difference $\varepsilon =\varphi_{L}-\varepsilon_{R}$ by setting $\varphi_{L}=-\varphi_{R}=\varphi /2$, the ROSI-induced phase difference $\theta_{R}=\theta_{R1}-\theta_{R2}$ with $\theta_{R1}=\theta_{R}$ and $\theta_{R2}=0$, magnetic flux ϕ, the configuration of the dots’ energy levels $\left(\varepsilon_{1},\varepsilon_{2}\right)$, and the interdot coupling strength $t_{c}$ at zero-temperature (T=0). Relevant constants are chosen to be $\hbar =e=k_{B}=1$. We consider the case that the left and right superconductors are made of the same material and couple to the DQDs with equal strengths, i.e., $\Delta_{L}=\Delta_{R}=\Delta$ and $\Gamma_{L}=\Gamma_{R}=\Gamma$. The superconducting energy gap is fixed to be Δ≡1 as the energy unit, and the coupling strength between the DQDs and the superconductor leads is chosen as Γ=0.1Δ throughout the paper.

### 3.1. Magnetic Flux ϕ=0

We first present in Figure 1 the supercurrent as a function of the superconducting phase difference φ in the absence of magnetic flux (ϕ=0) for different values of the RSOI-induced phase factor $\theta_{R}$ and dots’ energy levels $\left(\varepsilon_{1},\varepsilon_{2}\right)$. As is indicated by the black solid line Figure 1a in which $\theta_{R}=0$ and the dots’ levels configuration (0,0), the supercurrrent $J_{c}$ versus φ obeys a sine-shaped relation with a oscillation period of 2π, and it is zero at φ=nπ with integer n=1,2,… Such a sinφ-like line-shape holds unchanged when the dots’ energy levels are tuned away from the leads’ chemical potentials μ=0, as is indicated by the black solid lines in Figure 1c,d for the cases of (0.2,−0.2) and (0.1,−0.3), respectively. Accordingly, the positive and negative supercurrents are of the same magnitude, and the diode efficiency is zero. In the presence of finite RSOI-induced $\theta_{R}$, however, the $J_{c}∼\varphi$ relation depends on the dots’ energy level configuration $\left(\varepsilon_{1},\varepsilon_{2}\right)$. When both of the two dots’ energy levels are aligned to the leads’ chemical potential, i.e., a configuration of (0,0) in Figure 1b, the absolute value of either positive or negative supercurrents is simultaneously suppressed with increasing $\theta_{R}$ as compared to the black solid line for $\theta_{R}=0$. Moreover, the suppression of the negative supercurrent by the RSOI-induced phase factor $\theta_{R}$ is more obvious than that of the positive one, resulting in an asymmetric $J_{c}$ and SDE. In the case of the dots’ level (0.2,−0.2) in Figure 1c, the absolute values of the positive and negative supercurrents are individually suppressed and enhanced, which is different from the case of (0,0) in Figure 1b. As for the configuration of (0.1,−0.3) in Figure 1d, however, the magnitude of the positive supercurrent remains almost unchanged regardless of the value of $\theta_{R}$, whereas that of the negative supercurrent is obviously enhanced with increasing $\theta_{R}$ from 0 to π/2. In both of the three dots’ levels configurations, the oscillation period of $J_{c}$ with respect to φ remains 2π. Another important result brought about by $\theta_{R}$ is that $J_{c}\left(\phi =n\pi \right)\neq 0$, which is consistent with previous work [36]. This is because the presence of $\theta_{R}$ induces an additional left–right phase difference, which drives the supercurrent even under the condition of zero superconducting phase difference.

**Figure 2:**
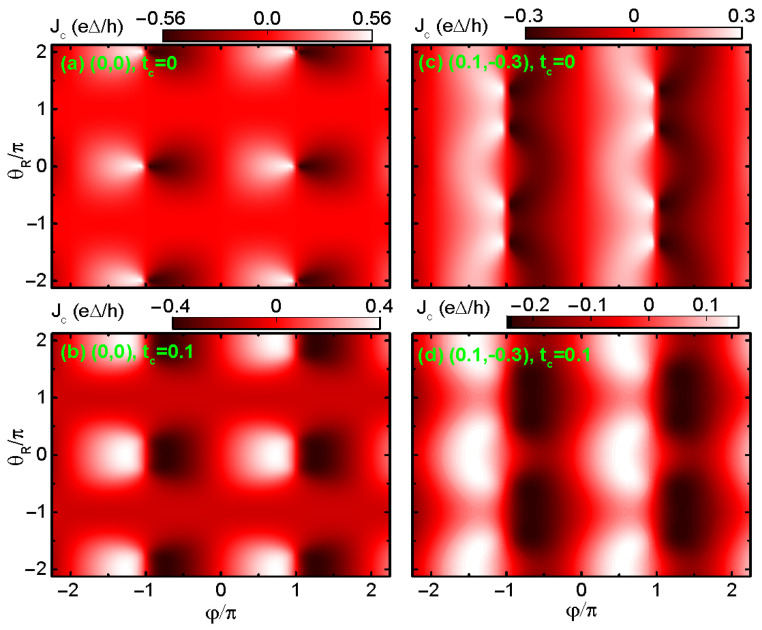
Contour plots of the supercurrent *J* versus supercoduncting phase φ and RSOI-induced phase $\theta_{R}$ for different values of $\left(\varepsilon_{1},\varepsilon_{2}\right)$ and $t_{c}$ in the absence of magnetic flux ϕ=0. Panels (**a**,**b**) are for the dots’ energy levels of (0, 0) with $t_{c}=0$ and 0.1, respectively. Panels (**c**,**d**) are for the dots’ energy levels of (0.1, −0.3) with $t_{c}=0$ and 0.1, respectively.

Since the behaviors of $J_{c}$ in the cases of dots’ energy levels configurations of (0.2,−0.2) and (0.1,−0.3) are essentially identical, we only show the results of (0,0) and (0.1,−0.3) in the following. Figure 2 shows that the supercurrent is a 2π-period function of both superconducting phase difference φ and $\theta_{R}$ regardless of the values of $\left(\varepsilon_{1},\varepsilon_{2}\right)$ and $t_{c}$. For (0,0) and $t_{c}=0$ in Figure 2a, and the $J_{c}∼\varphi$ line-shape for $\theta_{R}=0$ is the same as the black solid line in Figure 1b, which is characterized by the sinφ-like curve and equal positive and negative maxima. In the presence of RSOI, $J_{c}$ oscillates with respect to $\theta_{R}$ with a period of 2π. Different from usual oscillation, the RSOI-induced phase factor $\theta_{R}$ changes only the amplitude of $J_{c}$, leaving its sign unchanged. This holds true regardless of the value of $t_{c}$ and dots’ levels. In the presence of finite interdot coupling $t_{c}=0.1$, the absolute values of the positive and negative supercurrent are different from each other due to the RSOI-induced phase factor $\theta_{R}$, and the SDE arises. In particular, the supercurrent can be suppressed to zero around $\theta_{R}=\pm \pi$, and the positive critical current $J_{c+}=0$, accordingly. Now, the diode efficiency reaches its maximum, i.e., η=−2, which is a perfect diode phenomenon. For the case of (0.1,−0.3) and $t_{c}=0$, the behaviors of $J_{c}$ in Figure 2c essentially resemble those of Figure 2a, and no diode effect emerges due to the symmetric positive and negative supercurrents. For $t_{c}=0.1$ in Figure 2d, the phase of the positive supercurrent leads π more than that of the negative supercurrent, and then $J_{c}$ becomes obviously asymmetric with respect to φ.

**Figure 3:**
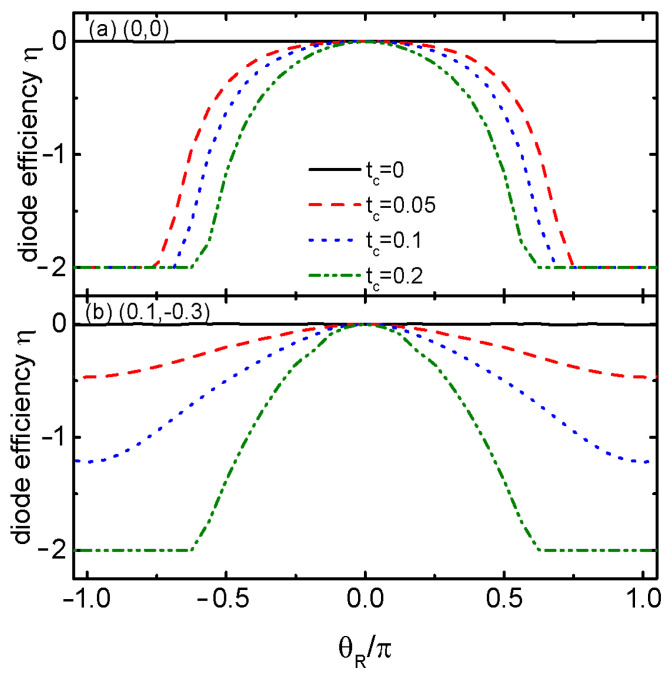
Diode efficiency η as a function of RSOI-induced phase factor $\theta_{R}$ for dots’ energy levels configurations of (0,0) in (**a**), and (0.1,−0.3) in (**b**) with indicated values of $t_{c}$.

Figure 3 shows the diode efficiency η varying with RSOI-induced phase factor $\theta_{R}$ for different dots’ energy levels configurations and interdot coupling strength $t_{c}$. In the case of the dots’ energy levels configuration (0,0), the diode efficiency η=0 when the two dots are decoupled from each other ($t_{c}=0$) as is indicated by the black solid line in Figure 3a. This is because now, the supercurrent is related to the superconducting phase difference by a sinusoidal-like function, and the absolute values of the positive and negative supercurrents are of the same magnitude. When the two QDs are hybridized to each other by even if very weak $t_{c}$, an SDE emerges on condition of finite $\theta_{R}$. Interestingly, the diode efficiency can reach its maximum −2 by the combined action of $t_{c}$ and $\theta_{R}$, which means that there is only a negative critical supercurrent in the absence of positive one. The perfect SDE with η=−2 may emerge in a rather large $\theta_{R}$ and is more likely to happen for larger values of $t_{c}$. In the case of (0.1,−0.3) as shown in Figure 3b, the diode efficiency is still zero for $t_{c}=0$ (black solid line), and it becomes negative when $t_{c}>0$. Compared to the case of (0,0) in Figure 3a, the diode efficiency for (0.1,−0.3) can reach −2 only under strong enough interdot coupling, for example, $t_{c}=0.15$ and 0.2 in Figure 3b. In the present paper, we add the RSOI-induced phase factor $\theta_{R}$ in the coupling strengths between the dots and the left lead $t_{Li\sigma }$, and the diode efficiency is negative. If $\theta_{R}$ is added in $t_{Ri\sigma }$, the diode efficiency should reverse its sign. Importantly, the magnetic flux may also reverse the sign of the diode efficiency, providing an efficient way of manipulating the SDE, as shown in the following.

### 3.2. Finite Magnetic Flux ϕ≠0

**Figure 4:**
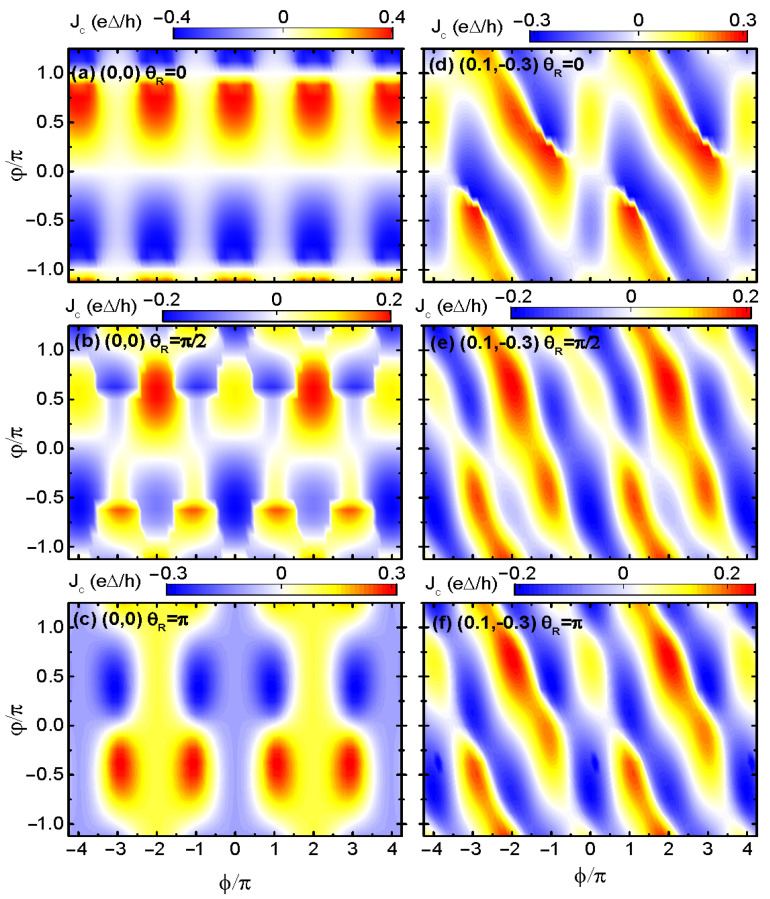
Contour plots of the supercurrent varying with respect to φ and ϕ with dots’ energy levels configuration (0,0) in (**a**–**c**) and (0.1,−0.3) in (**d**–**f**). The interdot coupling strength is fixed as $t_{c}=0.1\Delta$, and different values of $\theta_{R}$ are shown in the figures.

Figure 4 presents the supercurrent as a function of the superconducting phase difference φ and magnetic flux phase ϕ for different values of RSOI-induced phase $\theta_{R}$. When both of the two dots’ energy levels are in resonance with the leads’ fermi levels ($\varepsilon_{1}=\varepsilon_{2}=\mu$), the supercurrent is a 2π-period function of both φ and ϕ in the case of $\theta_{R}=0$, as shown in Figure 4a. The supercurrent oscillates with φ as a sine-like function, which is similar to the case in Figure 1b. Under this condition, the magnetic flux changes only the magnitude of the supercurrent but not its sign. Accordingly, the supercurrents flowing in positive and negative directions are equal to each other, and the SDE cannot occur. In the cases of dots’ levels (0,0), $\theta_{R}=\pi /2$ and $\theta_{R}=\pi$ as shown individually in Figure 4b,c, the period of $J_{c}$ with respect to ϕ becomes 4π, and that with φ remains 2π. Moreover, now both the magnitude and sign of the asymmetric supercurrent oscillate with ϕ, by which the SDE is adjustable. As for the case of the dot’s energy levels configuration of (0.1,−0.3) presented in Figure 4d–f, the supercurrent is a 2π- and 4π-period function of φ and ϕ, respectively. Similar to the case of (0,0) in Figure 4a–c, the supercurrent is anti-symmetric with respect to ϕ for $\theta_{R}=0$, and the SDE is absent. Whereas in the cases of $\theta_{R}=\pi /2$ and π in Figure 4e,f, the supercurrent is asymmetric with respect to φ, resulting in the SDE. The dependence of the supercurrent on the dots’ levels configuration and magnetic flux is consistent with the results in Ref. [43].

**Figure 5:**
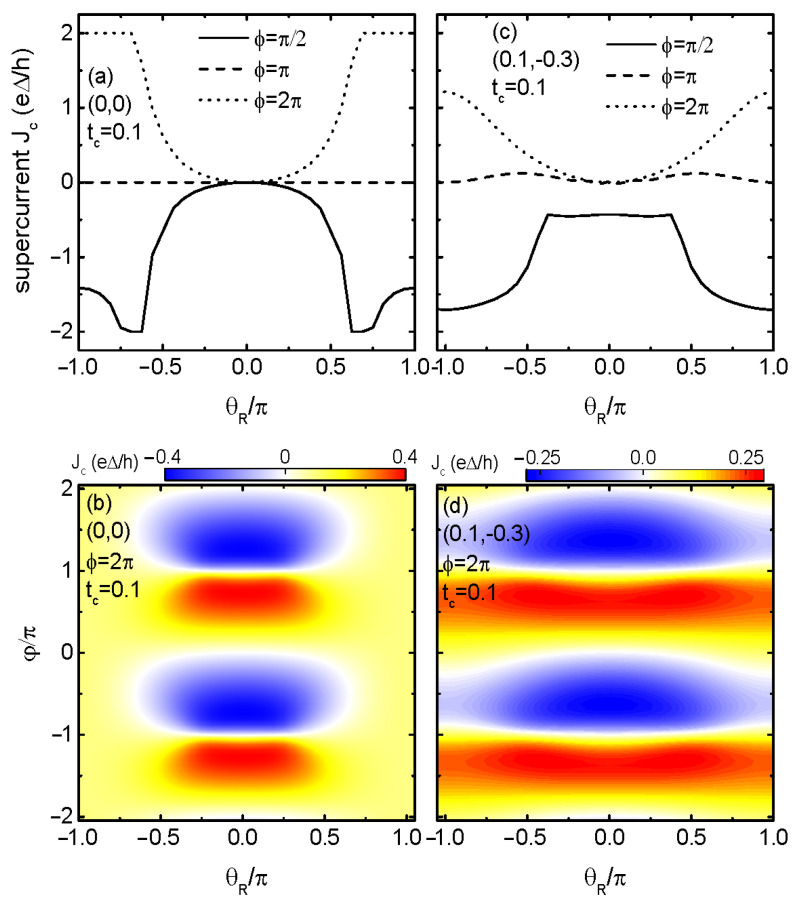
Supercurrent versus RSOI-induced phase factor $\theta_{R}$ for dots’ energy levels configuration (0, 0) in panel (**a**), and (0.1,−0.3) in panel (**c**). Panels (**b**,**d**) are contour plots for the supercurrent as a function of $\theta_{R}$ and superconducting phase difference ϕ for (0,0) and (0.1,−0.3), respectively. Other parameters are listed in the corresponding figure.

The black solid line in Figure 5a shows that for a relative small phase factor induced by the magnetic flux ϕ=π/2, the diode efficiency η essentially resembles that for ϕ=0 in Figure 3a indicated by the blue dotted line. It is zero at $\theta_{R}=0$ and becomes negative in the presence of finite $\theta_{R}$. Different from the case of ϕ=0 in Figure 3a, η>−2 at $\theta_{R}=\pm \pi$ for ϕ=π/2 in Figure 5a, and it may reach the value of −2 at a narrow regimes of $\theta_{R}$. When the magnetic flux phase factor ϕ=π, the diode efficiency is zero for all the values of $\theta_{R}$ due to the oscillation of the supercurrent with respect to the magnetic flux. Importantly, the diode efficiency becomes positive when ϕ=2π, as shown by the dot line in Figure 5a. Compared to the blue dot line in Figure 3a for $\theta_{R}=0$, the diode efficiency obeys the relation of $\eta \left(\theta_{R}\right)=-\eta \left(\theta_{R}+2\pi \right)$. Figure 5b shows that the supercurrent remains as a 2π-period function with respect to either superconducting phase difference φ or RSOI-induced phase factor $\theta_{R}$. The supercurrent is quite asymmetric with respect to φ and induces the SDE accordingly. When the value of the RSOI-induced phase factor is $0.7\pi ≃|\theta_{R}|\leq \pi$, the supercurrent is positive regardless of the value of φ, resulting in η=2, as shown in Figure 5a. If the dots’ energy levels are arranged in the configuration of (0.1,−0.3), the dependence of the diode efficiency in Figure 5c and the supercurrent in Figure 5d on the magnetic flux are similar to the case of (0,0). Figure 5 indicates that the magnetic flux provides an efficient means of adjusting both the magnitude and sign of the diode efficiency of the SDE.

**Figure 6:**
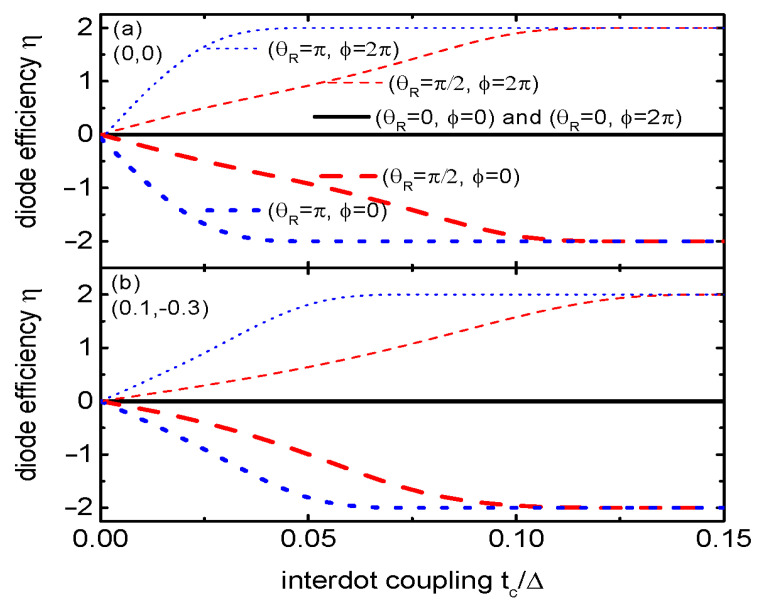
Diode efficiency η as a function of the interdot coupling $t_{c}$ and different values of ϕ and $\theta_{R}$. In panels (**a**,**b**), the dots’ energy levels are individually fixed at (0,0) and (0.1,−0.3).

Figure 6 presents the diode efficiency η versus interdot coupling strength $t_{c}$ for different values of RSOI-induced phase factor $\theta_{R}$ and magnetic flux ϕ. Under the conditions of dots’ energy levels (0,0) and ϕ=0 as indicated by the thin lines in Figure 6a, the diode efficiency is negative in the presence of finite RSOI, and its absolute increases with increasing $t_{c}$. For large enough values of $t_{c}$, the diode efficiency value becomes η=−2, indicating that there is only a negative supercurrent. These results are in agreement with those in Figure 3a. When the phase factor induced by the magnetic flux penetrating through the system is θ=2π, the diode efficiency becomes positive and approaches η=2, indicating that there is only a positive supercurrent flowing through the system. This result is also in consistent with that in Figure 5a. In the case of dots’ level configuration of (0.1,−0.3) as shown in Figure 6b, the behaviors of η resemble those of (0,0). But now $\eta \left(\theta_{R}\right)\neq -\theta \left(\theta_{R}+2\pi \right)$, which is different from that in Figure 6a. The reason is that the supercurrent’s period in the case of (0.1,−0.3) is different from that of (0,0) as shown in Figure 4. Since the interdot coupling strength $t_{c}$ relies on the distance between the two QDs, which are materials for the tunnel junction separating the two dots or gate voltages applied on the dots, adjusting the diode efficiency via $t_{c}$ provides a rich way of manipulating the SDE. Finally, we briefly discuss the value of the RSOI-induced phase factor $\theta_{R}$, which is given by $\theta_{R}=\pm k_{R}L$ with $k_{R}=\alpha m^{*}/\hbar^{2}$ and *L* the length of the QD. The quantity α is the RSOI strength, which may reach about $\alpha =1.16\times 10^{-11}$ eVm in a typical InSb with an effective mass of electrons $m^{*}=0.015m_{0}$. The value of $\theta_{R}$ then may reach about π for the length of the QD L≈300 nm, which is realizable in experiments [20,21,45,46,47].

## 4. Summary

In summary, the properties of the supercurrent and the SDE in a system consisting of semiconductor DQDs arranged in a parallel configuration and coupled to the left and right superconductors are investigated theoretically in the framework of the nonequilibrium Green’s function technique. Our results show that the phase factor induced by the RSOI in the host materials of the QDs provides an additional driving force for the supercurrent and makes the system left/right asymmetric. As a result, the magnitude of the positive and negative critical supercurrents may be different from each other, which leads to the SDE due to the combined effects of the RSOI-induced phase factor, dots’ energy levels, interdot coupling strength, and the magnetic flux penetrating through the system. More importantly, both the sign and amplitude of the diode efficiency can be adjusted by the magnetic flux, which may be useful in regulating superconductor-based functional devices. Compared to previous work regarding the SDE, here, the critical current can be totally suppressed to zero in one direction and allow the flow of a finite current in the opposite direction, resulting in the perfect diode effect. The SDE in the present manuscript originates essentially from the combined effects of the RSOI and the magnetic flux, which is quite different from some previous setups. This may reduce experimental requirements, and the device is realizable within current nanofabrication techniques. The SDE, which is useful in superconductor devices, and the structure can be further optimized by applying magnetic fields at the *x* or *y* directions as well as inserting the two QDs in one arm of the ring.

## Data Availability

All data included in this study are available upon request by contact with the corresponding author.

## References

[1] Josephson B.D. (1962). Possible new effects in superconductive tunnelling. Phys. Lett..

[2] Josephson B.D. (1974). The discovery of tunnelling supercurrents. Rev. Mod. Phys..

[3] Makhlin Y., Schön G., Shnirman A. (2001). Quantum-state engineering with Josephson-junction devices. Rev. Mod. Phys..

[4] Tafuri F. (2019). Fundamentals and Frontiers of the Josephson Effect.

[5] Blais A., Grimsmo A.L., Girvin S.M., Wallraff A. (2021). Circuit quantum electrodynamics. Rev. Mod. Phys..

[6] Koren G., Eyal A., Iomin L., Nitzav Y. (2021). Observation of Josephson-like Tunneling Junction Characteristics and Positive Magnetoresistance in Oxygen Deficient Nickelate Films of Nd_0.8_Sr_0.2_NiO_3-δ_. Materials.

[7] Braginski A.I. (2019). Superconductor electronics: Status and outlook. J. Supercond. Nov. Magn..

[8] Linder J., Robinson J.W. (2015). Superconducting spintronics. Nat. Phys..

[9] Nadeem M., Fuhrer M.S., Wang X. (2023). The superconducting diode effect. Nat. Rev. Phys..

[10] Hu J.P., Wu C.J., Dai X. (2007). Proposed Design of a Josephson Diode. Phys. Rev. Lett..

[11] Ando F., Miyasaka Y., Li T., Ishizuka J., Arakawa T., Shiota Y., Moriyama T., Yanase Y., Ono T. (2020). Observation of superconducting diode effect. Nature.

[12] Ideue T., Iwasa Y. (2020). One-way supercurrent achieved in an electrically polar film. Nature.

[13] Jiang K., Hu J. (2022). Superconducting diode effects. Nat. Phys..

[14] Pal B., Chakraborty A., Sivakumar P.K., Davydova M., Gopi A.K., Pandeya A.K., Krieger J.A., Zhang Y., Date M., Ju S. (2022). Josephson diode effect from Cooper pair momentum in a topological semimetal. Nat. Phys..

[15] Fulton T.A., Dynes R.C. (1970). Current-Phase Relations in Superconducting Bridges. Phys. Rev. Lett..

[16] Fulton T.A., Dunkleberger L.N., Dynes R.C. (1972). Quantum Interference Properties of Double Josephson Junctions. Phys. Rev. B.

[17] Sivakov A.G., Turutanov O.G., Kolinko A.E., Pokhila A.S. (2018). Spatial Characterization of the Edge Barrier in Wide Superconducting Films. Low Temp. Phys..

[18] Lyu Y.Y., Jiang J., Wang Y.L., Xiao Z.L., Dong S., Chen Q.H., Milošević M.V., Wang H., Divan R., Pearson J.E. (2021). Superconducting Diode Effect via Conformal-Mapped Nanoholes. Nat. Commun..

[19] Hou Y., Nichele F., Chi H., Lodesani A., Wu Y., Ritter M.F., Haxell D.Z., Davydova M., Ilić S., Glezakou-Elbert O. (2023). Ubiquitous Superconducting Diode Effect in Superconductor Thin Films. Phys. Rev. Lett..

[20] Baumgartner C., Fuchs L., Costa A., Picó-Cortés J., Reinhardt S., Gronin S., Gardner G.C., Lindemann T., Manfra M.J., Junior P.F. (2022). Effect of Rashba and Dresselhaus Spin-Orbit Coupling on Supercurrent Rectification and Magnetochiral Anisotropy of Ballistic Josephson Junctions. J. Phys. Condens. Matter.

[21] Amundsen M., Linder J., Robinson J.W.A., Žutić I., Banerjee N. (2024). Colloquium: Spin-orbit effects in superconducting hybrid structures. Rev. Mod. Phys..

[22] Yuan N.F.Q., Fu L. (2022). Supercurrent diode effect and finite-momentum superconductors. Proc. Natl. Acad. Sci. USA.

[23] Souto R.S., Leijnse M., Schrade C. (2022). Josephson Diode Effect in Supercurrent Interferometers. Phys. Rev. Lett..

[24] Gupta M., Graziano G.V., Pendharkar M., Dong J.T., Dempsey C.P., Palmstrøm C., Pribiag V.S. (2023). Gate-tunable superconducting diode effect in a three-terminal Josephson device. Nat. Commun..

[25] Coraiola M., Svetogorov A.E., Haxell D.Z. (2024). Flux-Tunable Josephson Diode Effect in a Hybrid Four-Terminal Josephson Junction. ACS Nano.

[26] Zwolak J.P., Taylor J.M. (2023). Colloquium: Advances in automation of quantum dot devices control. Rev. Mod. Phys..

[27] Martín-Rodero A., Yeyati A.L. (2011). Josephson and Andreev transport through quantum dots. Adv. Phys..

[28] Sun Q.F., Wang J., Lin T.H. (1999). Photon-assisted andreev tunneling through a mesoscopic hybrid system. Phys. Rev. B.

[29] Sun Q.F., Wang J., Lin T.H. (2000). Control of the supercurrent in a mesoscopic four-terminal Josephson junction. Phys. Rev. B.

[30] Zhu Y., Sun Q.F., Lin T.H. (2001). Andreev bound states and the *π*-junction transition in a superconductor/quantum-dot/superconductor system. J. Phys. Condens. Matter.

[31] Buitelaar M.R., Nussbaumer T., Schönenberger C. (2002). Quantum Dot in the Kondo Regime Coupled to Superconductors. Phys. Rev. Lett..

[32] Droste S., Andergassen S., Splettstoesser J. (2012). Josephson current through interacting double quantum dots with spin-orbit coupling. J. Phys. Condens. Matter.

[33] Cheng S.G., Sun Q.F. (2008). Josephson current transport through T-shaped double quantum dots. J. Phys. Condens. Matter.

[34] Hofstetter L., Csonka S., Nygard J., Schönenberger C. (2009). Cooper pair splitter realized in a two-quantum-dot Y-junction. Nature.

[35] Sun Y.F., Mao Y., Sun Q.F. (2023). Design of Josephson diode based on magnetic impurity. Phys. Rev. B.

[36] Debnath D., Dutta P. (2024). Gate-tunable Josephson diode effect in Rashba spin-orbit coupled quantum dot junctions. Phys. Rev. B.

[37] Mao Y., Yan Q., Zhuang Y.C., Sun Q.F. (2024). Universal Spin Superconducting Diode Effect from Spin-Orbit Coupling. Phys. Rev. Lett..

[38] Xu L.T., Li X.Q., Sun Q.F. (2017). Majorana dc Josephson current mediated by a quantum dot. J. Phys. Condens. Matter.

[39] Chi F., Jia Q.S., Liu J., Gao Q.G., Yi Z.C., Liu L.M. (2023). Enhancement of the Josephson Current in a Quantum Dot Connected to Majorana Nanowires. Nanomaterials.

[40] Zhang H.R., Sun L.L., Liu J. (2023). Josephson dc Current through T-Shaped Double-Quantum-Dots Hybridized to Majorana Nanowires. Coatings.

[41] Gao Y.M., Zhang X.Y. (2023). Tunable Josephson Current through a Semiconductor Quantum Dot Hybridized to Majorana Trijunction. Coatings.

[42] Gao Y.M., Xiao H., Jiang M.H., Chi F., Yi Z.C., Liu L.M. (2024). Josephson Diode Effect in Parallel-Coupled Double-Quantum Dots Connected to Unalike Majorana Nanowires. Nanomaterials.

[43] Pan H., Lin T.H. (2006). Control of the supercurrent through a parallel-coupled double quantum dot system. Phys. Rev. B.

[44] Pan H., Lin T.H. (2007). Tunable supercurrent in a parallel double quantum dot system. Eur. Phys. J. B.

[45] Deacon R.S., Oiwa A., Sailer J., Baba S., Kanai Y., Shibata K., Hirakawa K., Tarucha S. (2015). Cooper pair splitting in parallel quantum dot Josephson junctions. Nat. Commun..

[46] Debbarma R., Aspegren M., Boström F.V., Lehmann S., Dick K., Thelander C. (2022). Josephson Current via Spin and Orbital States of a Tunable Double Quantum Dot. Phys. Rev. B.

[47] Debbarma R., Tsintzis A., Aspegren M., Souto R.S., Lehmann S., Dick K., Leijnse M., Thelander C. (2023). Josephson Junction *π* − 0 Transition Induced by Orbital Hybridization in a Double Quantum Dot. Phys. Rev. Lett..

[48] Sun Q.F., Wang J., Guo H. (2005). Quantum transport theory for nanostructures with Rashba spin-orbital interaction. Phys. Rev. B.

[49] Sun Q.F., Xie X.C. (2006). Bias-controllable intrinsic spin polarization in a quantum dot: Proposed scheme based on spin-orbit interaction. Phys. Rev. B.

[50] Chi F., Li S.S. (2006). Spin-polarized transport through an Aharonov-Bohm interferometer with Rashba spin-orbit interaction. J. Appl. Phys..

[51] Pan H., Cui Y.M., Wang H.L., Wang R.M. (2011). Spin-polarized Andreev reflection and spin accumulation in a quantum-dot Aharonov-Bohm interferometer with spin-orbit interaction effects. J. Appl. Phys..

[52] Reinhardt S., Ascherl T., Costa A., Berger J., Gronin S., Gardner G.C., Lindemann T., Manfra M.J., Fabian J., Kochan D. (2024). Link between supercurrent diode and anomalous Josephson effect revealed by gate-controlled interferometry. Nat. Commun..

[53] Garcia-Merino J.A., Mercado-Zuniga C., Hernandez-Acosta M.A., Aguilar-Pérez L.A., Villanueva-Fierro I., Hevia S.A., Torres-Torres C. (2021). Magnetic frequency identification by quantum interference in magnetoplasmonic carbon/metal nanostructures. Mat. Sci. Eng. B.

